# A Review of the Management of Nephrolithiasis in Autosomal Dominant Polycystic Kidney Disease

**DOI:** 10.7759/cureus.94642

**Published:** 2025-10-15

**Authors:** Rakhul Raveendran, Vinodh Murali, Herman Fernando

**Affiliations:** 1 Urology, University Hospitals of North Midlands, Stoke-on-Trent, GBR

**Keywords:** adpkd, autosomal dominant polycystic kidney disease (adpkd), flexible ureteroscopy, nephrolithiasis, pcnl, renal cyst, rirs, shock wave lithotripsy, urolithiasis

## Abstract

Autosomal dominant polycystic kidney disease (ADPKD) is a chronic hereditary disorder and a major cause of end-stage renal disease (ESRD). Urolithiasis is a frequent complication in ADPKD and may contribute to the accelerated decline in renal function. The presence of complex calyceal anatomy, impaired renal function, and associated comorbidities makes stone management particularly challenging in this population.

We conducted a narrative review of the literature to explore the underlying reasons for the high incidence of nephrolithiasis in ADPKD, as well as current approaches to management. A targeted literature search was performed in MEDLINE, EMBASE, Google Scholar, and Web of Science for articles published up to February 2025 using the keywords "ADPKD", "nephrolithiasis", "kidney stones," "urolithiasis," "management", and "treatment". Only English-language articles involving human subjects were considered. Original studies, meta-analyses, narrative, and systematic reviews relevant to nephrolithiasis in ADPKD were included. Conference abstracts, editorials, and non-peer-reviewed sources were excluded. Both medical and surgical treatment modalities were evaluated, with attention to reported success rates and complication profiles.

Although this is a narrative review and no formal risk of bias assessment was performed, emphasis was placed on including high-quality studies and widely cited literature. Key findings were synthesized thematically to provide an overview of current understanding and management of nephrolithiasis in ADPKD. Small sample sizes and significant heterogeneity in study design, patient selection, and treatment protocols limit available evidence. While the general principles of stone management are similar to those in non-ADPKD patients, therapeutic decisions must be tailored to stone size, location, composition, renal function, and anatomical considerations. Based on the literature, we propose a treatment algorithm to support clinical decision-making. Standardization of management strategies in ADPKD remains an unmet need. Larger, well-designed studies are required to establish evidence-based guidelines for optimal care.

## Introduction and background

Autosomal dominant polycystic kidney disease (ADPKD) is an inherited systemic disorder with an estimated incidence ranging from one in 400 to one in 1000 live births. Two genetic subtypes have been identified. The more common form is caused by mutations in the PKD1 gene, located on the short arm of chromosome 16, which encodes polycystin-1. A less frequent variant results from mutations in the PKD2 gene on the long arm of chromosome 3, encoding polycystin-2.

The clinical course of ADPKD varies by genotype. PKD1-related disease typically manifests earlier, progresses more rapidly, and leads to end-stage renal disease (ESRD) in the fifth or sixth decade of life. By contrast, PKD2-related disease is characterized by slower progression and later onset of ESRD. Disease severity also appears to be greater when maternally inherited, with earlier and more aggressive manifestations reported across generations [1].

Nephrolithiasis is significantly more common in patients with ADPKD than in the general population, with reported incidence ranging from 8% to 36% [2-5]. The relative risk of stone formation is estimated to be 2-10 times higher in ADPKD [3,6], and prevalence is similar in men and women [3]. The mean age at presentation is approximately 39 ± 14 years for symptomatic cases and 42 ± 12 years for asymptomatic cases [3]. Between 25% and 50% of patients present with symptoms [3,7]. Significantly, nephrolithiasis may accelerate the decline of renal function and increase the risk of recurrent infections in polycystic kidneys [8-10].

## Review

Pathophysiology of stone formation in PKD

The increased incidence of urolithiasis in polycystic kidneys can be attributed to a combination of anatomical and metabolic factors characteristic of these kidneys.

Anatomical Factors

Cyst expansion in ADPKD leads to distortion of the collecting system and obstruction of normal urine flow, resulting in urinary stasis, which is a key contributing factor to stone formation in these patients [11,12]. Both the number and size of cysts are significantly greater in ADPKD patients with stones compared to those without [12]. As the disease progresses, distortion of the collecting system increases, further promoting urinary stasis and creating a favorable environment for stone formation [11,12]. This distortion also alters the medullary architecture and impairs tubular function, particularly ammonia handling and excretion, resulting in a persistently low urinary pH [13].

Metabolic Factors

Seminal studies by Torres et al., Grampsas et al., and others have demonstrated two main metabolic abnormalities in the urine of ADPKD patients: hypocitraturia and low urinary pH [3,12,13].

Interestingly, the common metabolic abnormalities observed in non-PKD stone formers, such as hypercalciuria, hyperoxaluria, and hyperuricosuria, are uncommon in this group [5,11]. The metabolic profile in ADPKD explains the typical stone types found: uric acid stones (primarily due to low urinary pH rather than hyperuricemia or increased uric acid excretion) and calcium oxalate monohydrate stones (linked to hypocitraturia).

Low urinary pH is believed to result from defective ammonia excretion in the distal tubules [5]. The precise cause of hypocitraturia remains unclear, although it is no longer attributed to distal renal tubular acidosis as once proposed. Additional contributing factors include low urinary levels of magnesium, phosphate, and potassium [5,7], as well as reduced urine volume-ADPKD patients typically produce up to 30% less urine than the general population.

Recognition of these pathophysiological mechanisms supports the widespread use of potassium citrate in managing nephrolithiasis in PKD. Potassium citrate is highly effective in conditions associated with ADPKD, including uric acid stones, hypocitraturia, calcium oxalate stones, and distal acidification defects [5,14].

Management of stones in PKD kidneys

Flank pain and hematuria are the most common manifestations of urolithiasis in ADPKD. However, these symptoms may result from a range of complications, including urolithiasis, cyst hemorrhage, cyst infection, and, rarely, cyst neoplasm. In some cases, the pain may be nonspecific [15]. Differentiating among these causes is crucial. Low back pain has also been reported more frequently in ADPKD patients with stones [16]. Around half of these patients are symptomatic, but the other half remain asymptomatic, and in them, renal function may deteriorate silently. Although the basic principles of stone management are similar to those in non-PKD patients, close monitoring and early intervention are essential to prevent complications and further decline in renal function in individuals with already impaired glomerular filtration.

Investigations

Routine blood and urine tests, assessment of renal function, and urine cultures should be performed in all patients. Imaging, however, plays the central role in management planning.

Abdominal Imaging

All standard imaging modalities can be used in PKD patients with stones, but each has specific limitations and advantages.

Plain X-ray kidney, ureter, and bladder: Plain X-ray is unreliable in PKD, as most stones are uric acid and radiolucent. Additionally, it cannot distinguish between parenchymal/cyst wall calcifications and calculi. Its role in modern clinical practice is very limited, and it should not be used as the sole investigation in suspected cases.

Ultrasonography: Ultrasound is a useful initial screening test, with a specificity of about 90%, but its sensitivity is as low as 24% [17]. It cannot reliably differentiate stones from calcifications and is highly operator-dependent. Multiple cysts often obscure visualization of calculi and distort acoustic windows [16]. While it may be helpful in experienced hands, variability and confounding factors such as cyst calcification reduce its reliability.

Computed tomography (CT) scan: CT offers the highest sensitivity and specificity for stone detection, both in the general population [16] and in ADPKD. In ADPKD, sensitivity and specificity are reported at 63% and 81%, respectively. CT not only detects stones but also provides detailed information about kidney anatomy, collecting system distortion, cyst-related compression, and associated complications such as hemorrhage, rupture, calcification, or neoplasm. It also evaluates extrarenal manifestations of the disease [18].

Dual-energy CT can further differentiate uric acid from calcium stones, guiding management [19]. CT gives valuable data on stone size, location, density, and calyceal anatomy, all critical for surgical planning. It is the gold standard for confirming stone clearance and for follow-up. New-generation CT scanners, with improved resolution and rapid acquisition, have transformed stone imaging. Where renal function allows, contrast CT is preferred, as it distinguishes cyst calcifications from stones and provides precise calyceal mapping.

Stone Analysis and Metabolic Evaluation

Stone analysis should be part of the diagnostic work-up, as it helps identify underlying pathology. Most stones in PKD patients are uric acid, with calcium oxalate monohydrate stones being less frequent [3,16]. Unusual stone types should raise suspicion of infection or an additional metabolic disorder.

Metabolic evaluation is advisable in all stone-forming PKD patients, as they have a high risk of recurrence. Early identification of correctable anomalies helps reduce morbidity. The most common findings include hypocitraturia, persistently low urinary pH, and reduced urine volume [7,13]. Most ADPKD patients have a urinary pH below 5.5, either at baseline or after an acid load, thereby excluding distal RTA [5,15,16]. These patients benefit from adequate hydration and alkalinization with oral potassium citrate, which prevents uric acid crystallization and reduces calcium oxalate supersaturation.

Surgical options for stone removal

Extracorporeal Shockwave Lithotripsy (ESWL) in ADPKD

Since the report by Delakas et al. [20], renal cysts are no longer considered a contraindication to ESWL [20-21]. Initial concerns about cyst hemorrhage were not substantiated in later studies [20-22]. Nonetheless, it is advisable to minimize the number of shocks delivered. Stone clearance rates after ESWL are consistently lower than in normal kidneys [20,21], mainly due to cyst-induced obstruction of the collecting system, which prevents fragment passage [21]. Reported clearance rates range from 0 to 80% [20-21,23], with better outcomes for pelvic stones than for calyceal stones [23].

Complications such as cyst hemorrhage, rupture, perirenal bleeding, and infection are rare in modern series when the procedure is properly performed [20-23]. The main limitation is incomplete clearance, often necessitating multiple ESWL sessions or adjunctive procedures [20]. Routine ureteric stenting is not required [24-25], although some authors recommend placing a DJ stent pre-procedure in PKD to prevent obstruction-related complications [20].

Even in non-PKD patients, ESWL is less effective in those with dense stones, long thin infundibula, acute infundibulopelvic angles, or high skin-to-stone distance, all factors commonly present in PKD. Therefore, ESWL success is limited, but it may still be useful for small, low-density stones in favorable anatomy or as an adjunct to percutaneous nephrolithotomy (PCNL)/retrograde intrarenal surgery (RIRS).

Flexible Ureterorenoscopy (RIRS)

The introduction of flexible ureteroscopes and miniaturized instruments has enabled retrograde access to the collecting system. However, in ADPKD, distorted calyceal anatomy and elongated infundibula make RIRS technically demanding. Residual fragments are unlikely to pass unless thoroughly dusted, particularly in lower-pole stones [26]. Most series report the need for multiple sittings to achieve clearance, with higher rates of emergency department visits compared to non-PKD patients [27]. Thus, RIRS was traditionally reserved for patients with low stone burden and favorable anatomy, with prior counselling about possible auxiliary procedures [23,26-28].

Over the last decade, advances in digital scopes, holmium/thulium lasers, access sheaths, and suction devices have improved outcomes. Despite limited data (the largest series: 11 patients [23,26,28-30]), reported success rates range from 73% to 100% after a single session. Most complications are minor, such as fever, hematuria, or pain. Xu et al. [31] reported shorter operating times, shorter hospital stays, and clearance rates of up to 85%. With ongoing innovations, RIRS is likely to become more widely applicable, offering better clearance than ESWL and less invasiveness than PCNL. ECIRS (combined antegrade and retrograde approach) is another promising option for complex stones [32].

PCNL in ADPKD

PCNL remains the most studied and established intervention for stones in ADPKD. Challenges include abnormal calyceal anatomy, elongated/narrow calyces, multiple cysts, thin parenchyma, and pre-existing renal impairment [6,9,10,14,29].

Initial puncture may be guided by fluoroscopy or ultrasound. Ultrasound is hampered by cyst interference, but it reduces the risk of inadvertent cyst puncture [23]. Fluoroscopy is more commonly used [6,10,14,29]. Distinguishing cyst fluid from urine during puncture confirmation can be difficult; methods include retrograde instillation of dye [10], saline [7,23], or contrast [33]. Cyst aspiration may also aid localization [14].

Choice of calyx depends on the stone location and calyceal width. For narrow calyces, another access should be chosen rather than over-dilating. Tracts may be dilated up to 28 Fr, with care to avoid parenchymal damage. Multiple punctures are possible but best avoided in marginal renal function. Supine PCNL has shown promising results, as demonstrated in the 2022 series by Choudhury et al. [34], which reported outcomes comparable to prone PCNL, with the added advantage of enabling endoscopic combined intrarenal surgery (ECIRS) [32].

Most series recommend nephrostomy tubes and DJ stents postoperatively [6,10,14,23,28,33]. Operating time, blood loss, and hospital stay are typically greater than for RIRS [28], reflecting the higher invasiveness and stone burden. Imaging (CT or nephrostogram) should confirm clearance before tube removal [6,10,14,28].

Reported success rates for PCNL range from 45% to 100% in different series, with auxiliary procedures required in up to 64% of cases. A systematic review by Kalatharan et al. highlighted heterogeneity in outcomes. At the same time, the only cohort study (Khorrami et al.) found no significant difference in clearance, renal damage, or blood loss between PKD and non-PKD patients [35].

**Figure 1:**
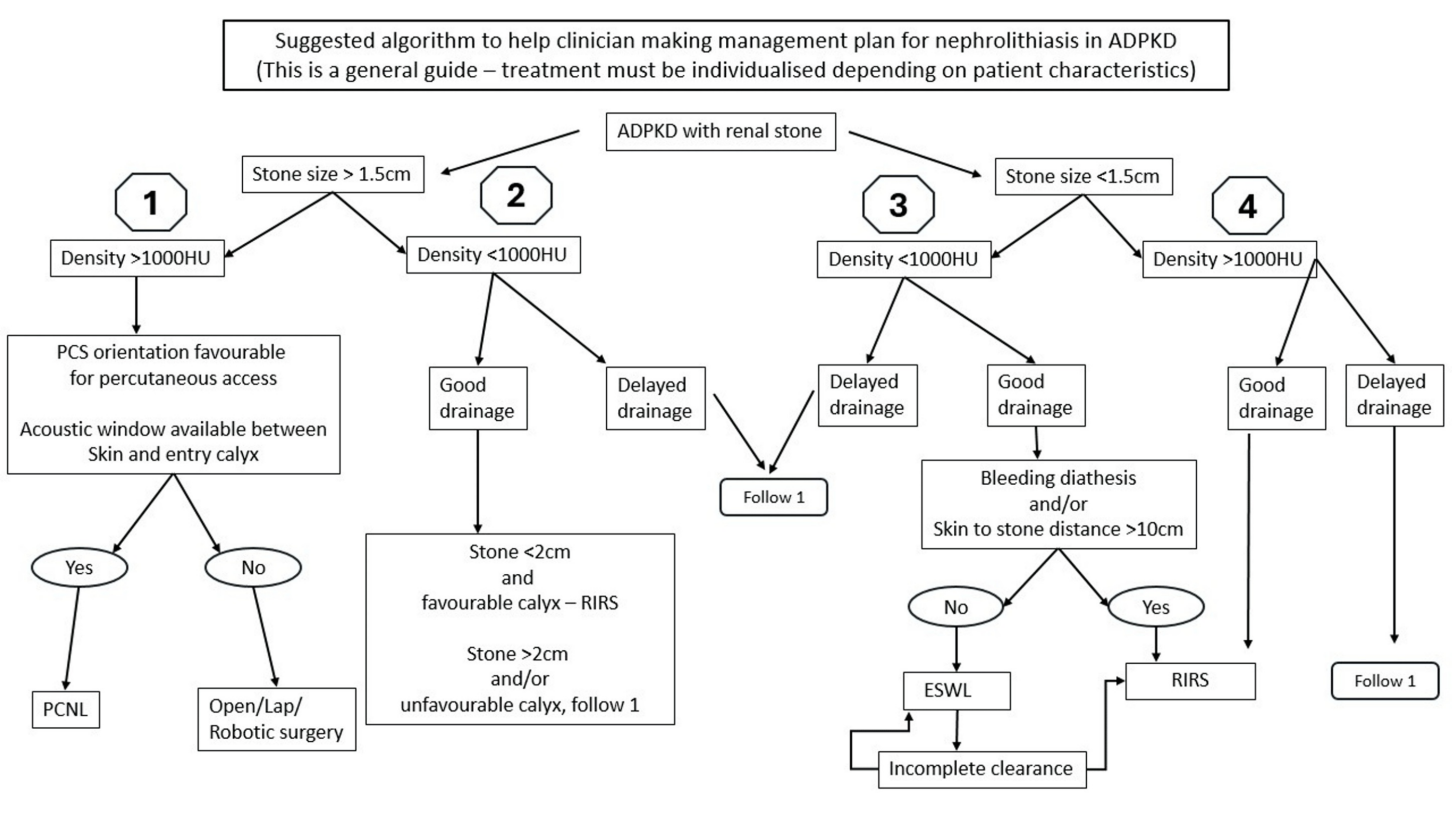
Algorithm for managing nephrolithiasis in ADPKD patients ADPKD: autosomal dominant polycystic kidney disease, HU: Hounsfield unit, PCS: pelvicalyceal system, PCNL: percutaneous nephrolithotomy, RIRS: retrograde intrarenal surgery, ESWL: extracorporeal shock wave lithotripsy

The success rates, specifically the stone-free rate, in various series are shown in Figure 1.

**Table 1:** Success rates in terms of stone-free rate following PCNL in various series Data are comparable with the stone clearance rates in non-PKD patients undergoing PCNL. PCNL: percutaneous nephrolithotomy, ESWL: extracorporeal shockwave lithotripsy, PKD: polycystic kidney disease

	Authors	Stone clearance (after first PCNL)	Stone clearance (after 2nd procedure PCNL/ESWL)
1	Al-Kandari et al., 2009 [10]	NA	89.4%
2	Umbreit et al., 2010 [14]	82%	100%
3	Srivastava et al., 2011 [6]	88%	96%
4	Baishya et al., 2012 [23]	67%	100%
5	Singh et al., 2013 [29]	82.1%	92.85%
6	Singh et al., 2019 [30]	83%	100%

Complications of PCNL in PKD Patients

Overall, complication rates in ADPKD are comparable to those in non-PKD patients. The main surgical complications reported in the literature are summarized below.

Bleeding requiring transfusion: The incidence ranges from 0% to 13% in most series [6,10,14,33], which is similar to the incidence in non-PKD PCNL cases (0-17.5%) [10]. All episodes were managed with transfusion, and none required angioembolization or open surgery. Nevertheless, PCNL in ADPKD should ideally be performed in centers with access to interventional radiology.

Postoperative fever due to cyst infection: This complication is relatively unique to ADPKD, with an incidence of 0-18%. Cultures may be sterile, and fever can be refractory to conventional antibiotics due to poor drug penetration into cysts. Ultrasound or CT can help identify infected cysts, which may require aspiration and targeted antibiotic therapy [8,29,36]. Long-term treatment with cyst-penetrating lipophilic antibiotics may occasionally be necessary. The 2025 Kidney Disease Improving Global Outcomes (KDIGO) clinical practice guidelines recommend four to six weeks of antibiotic treatment [37].

Deterioration of renal function: Available evidence does not suggest persistent renal function decline after PCNL in ADPKD. Renal function generally remains stable or improves [6,10]. In the series by Singh et al., three patients had transient worsening of renal function postoperatively, which resolved with conservative management.

Other complications: Rates of collecting system injury, visceral injury, thoracic complications, and neuromusculoskeletal events are comparable to those seen in non-PKD patients.

To summarize, PCNL in ADPKD achieves satisfactory stone-free rates with acceptable morbidity. While the approach differs slightly from conventional PCNL, and multiple procedures may be required for complete clearance, overall outcomes and complication rates are comparable to those of kidneys with normal anatomy. Therefore, PCNL should be considered a viable option for ADPKD patients with a significant stone burden.

Open/laparoscopic/robotic surgery: Historically, most ADPKD patients underwent open pyelolithotomy for stone removal, but this approach carried high morbidity. With the advent of minimally invasive techniques, open surgery is rarely required today. It may still be considered in patients with unfavorable anatomy or extensive stone burden.

Where expertise and resources are available, laparoscopic or robotic pyelolithotomy/ureterolithotomy can be an alternative. In non-salvageable kidneys with infection and stones, nephrectomy may be required. Laparoscopic nephrectomy in ADPKD is technically challenging, and open surgery is often preferred. Hand-assisted laparoscopic nephrectomy is another option in experienced centers, and, in fact, the latest KDIGO guideline recommends hand-assisted laparoscopy as the standard approach for nephrectomy in PKD patients [37].

Medical management, prevention, and follow-up of nephrolithiasis in ADPKD

Since most stones in ADPKD are composed of uric acid, and the dominant metabolic abnormalities include low urinary pH and hypocitraturia, medical therapy with potassium citrate may be considered. Dissolution therapy can be effective for small, asymptomatic stones, though the precise threshold stone size for intervention is not clearly defined.

In modern practice, potassium citrate is best used both as a preventive measure and as a treatment for small, non-obstructing uric acid calculi in cystic kidneys with preserved renal function. KDIGO guidelines (2025) recommend that patients should undergo 24-hour urinary testing for lithogenic risk factors, serial kidney imaging studies to assess their stone burden, and analysis of their kidney stones if feasible [37].

For stone prevention, recommendations for the general population with kidney stones are also applicable to ADPKD patients. The most important factor is maintaining a high fluid intake to ensure a urine output of 2.5 liters a day. This helps to reduce the risk of kidney stones by 60-80%. There is also a potential added advantage of reducing the kidney cyst growth by suppressing arginine vasopressin release, as shown in preclinical studies [38]. A healthy diet rich in fiber, fruits, and vegetables, while low in salt and animal protein, is recommended. In addition, specific dietary modifications can be made based on urinary lithogenic risk factors.

For follow-up, urinary studies should be individualized and repeated at one year and periodically thereafter, depending on the activity of stone formation. Additional follow-up kidney imaging should be individualized, with the preferred modality being a non-contrast low-dose CT [37].

We suggest an algorithm to help with clinical decision-making (Figure 1). This is only a general guide. It will need modification based on individual patient characteristics.

## Conclusions

Nephrolithiasis is a frequent and clinically significant complication in patients with ADPKD. Stone formation results from a combination of anatomical distortion caused by cysts and metabolic abnormalities such as hypocitraturia and persistently low urinary pH. Careful evaluation is required, as symptoms like flank pain and hematuria may also arise from other PKD-related complications. CT remains the most reliable imaging modality for diagnosis and surgical planning, while metabolic evaluation and stone analysis are essential to guide preventive strategies.

Treatment principles are broadly similar to those in non-PKD patients, but the distorted renal anatomy often makes management more challenging. Among surgical options, ESWL may be useful for selected patients with small stones and favorable anatomy, but clearance rates are limited. RIRS has become increasingly effective with technological advances, though multiple procedures are often required. PCNL remains the most established and reliable option for patients with a large stone burden, with success and complication rates comparable to non-PKD cases when performed in experienced centers. Open or laparoscopic surgery is now rarely required, reserved only for complex or non-salvageable cases.

Medical management, particularly with potassium citrate, plays an important role both in preventing recurrence and in managing small uric acid stones in patients with preserved renal function. Optimal management of nephrolithiasis in ADPKD requires a tailored approach that balances stone characteristics, renal function, and anatomical considerations. Early recognition, appropriate imaging, metabolic correction, and timely intervention can help prevent complications and preserve kidney function in this vulnerable patient population. However, the current evidence is largely derived from small retrospective series, and there remains a lack of large, high-quality prospective studies to guide practice and establish standardized treatment protocols.
